# Biotransformation of Silver Nanoparticles into Oro-Gastrointestinal Tract by Integrated In Vitro Testing Assay: Generation of Exposure-Dependent Physical Descriptors for Nanomaterial Grouping

**DOI:** 10.3390/nano11061587

**Published:** 2021-06-17

**Authors:** Catherine Carnovale, Daniela Guarnieri, Luisana Di Cristo, Isabella De Angelis, Giulia Veronesi, Alice Scarpellini, Maria Ada Malvindi, Flavia Barone, Pier Paolo Pompa, Stefania Sabella

**Affiliations:** 1 Istituto Italiano Di Tecnologia, Nanoregulatory Platform, Drug Discovery and Development Department, 16163 Genova, Italy; catherine.carnovale@gmail.com (C.C.); luisana.dicristo@iit.it (L.D.C.); 2 Dipartimento di Chimica e Biologia “A. Zambelli”, Università di Salerno, Via Giovanni Paolo II 132, Fisciano, 84084 Salerno, Italy; dguarnieri@unisa.it; 3 Research Centre for Biomaterials BIONAM, University of Salerno, Via Giovanni Paolo II 132, Fisciano, 84084 Salerno, Italy; 4 Istituto Superiore di Sanità (ISS), 00161 Rome, Italy; isabella.deangelis@iss.it (I.D.A.); flavia.barone@iss.it (F.B.); 5 Laboratory of Chemistry and Biology of Metals (CBM), University Grenoble Alpes/CNRS/CEA, 38000 Grenoble, France; giulia.veronesi@cea.fr; 6 ESRF, the European Synchrotron, 71 Av. des Martyrs, 38000 Grenoble, France; 7 Electron Microscopy Facility, Istituto Italiano di Tecnologia, Via Morego 30, 16163 Genova, Italy; alice.scarpellini@iit.it; 8 HiQ-Nano, s.r.l. Via Barsanti, Arnesano, 73010 Lecce, Italy; mariada.malvindi@hiqnano.com; 9 Nanobiointeractions & Nanodiagnostics, Istituto Italiano di Tecnologia (IIT), Via Morego 30, 16163 Genova, Italy; pierpaolo.pompa@iit.it

**Keywords:** consecutive dissolution test, human digestion, silver nanoparticles, oral exposure, bioavailability, grouping, exposure dependent physical descriptors

## Abstract

Grouping approaches of nanomaterials have the potential to facilitate high throughput and cost effective nanomaterial screening. However, an effective grouping of nanomaterials hinges on the application of suitable physicochemical descriptors to identify similarities. To address the problem, we developed an integrated testing approach coupling acellular and cellular phases, to study the full life cycle of ingested silver nanoparticles (NPs) and silver salts in the oro-gastrointestinal (OGI) tract including their impact on cellular uptake and integrity. This approach enables the derivation of exposure-dependent physical descriptors (EDPDs) upon biotransformation of undigested nanoparticles, digested nanoparticles and digested silver salts. These descriptors are identified in: size, crystallinity, chemistry of the core material, dissolution, high and low molecular weight Ag-biomolecule soluble complexes, and are compared in terms of similarities in a grouping hypothesis. Experimental results indicate that digested silver nanoparticles are neither similar to pristine nanoparticles nor completely similar to digested silver salts, due to the presence of different chemical nanoforms (silver and silver chloride nanocrystals), which were characterized in terms of their interactions with the digestive matrices. Interestingly, the cellular responses observed in the cellular phase of the integrated assay (uptake and inflammation) are also similar for the digested samples, clearly indicating a possible role of the soluble fraction of silver complexes. This study highlights the importance of quantifying exposure-related physical descriptors to advance grouping of NPs based on structural similarities.

## 1. Introduction

Among metal-based nanomaterials, nanosilver has emerged as the most commonly used, with applications spanning personal care (including sunscreen, cosmetics, antimicrobials and wound dressings), air or water treatment products, and food packaging [1,2,3,4,5]. Indeed, silver nanoparticles (AgNPs) are mostly used as antimicrobial packaging playing an important role in extending shelf-life of foods and reducing the risk of pathogens [6]. Due to the nature of these applications, the likelihood of increased ingestion of nanoparticulate silver (intentional or unintentional) is predicted to increase [7]. The development of regulations for their manufacture and use has struggled to keep pace due to the lack of exposure and hazard information, which requires data collection for each product individually. This process requires tremendous time and cost investment. A pragmatic alternative to testing each product individually must be sought to deliver a high-throughput and cost-effective screening solution [8]. Due to their predictive potential, grouping approaches and read-across schemes can be helpful in this framework [9]. The concept of grouping “like” substances, based on similarities in structural properties, has had great success in the area of chemical safety [8]. Despite attempts from highly active research projects [10], the transfer of these principals to the area of nanomaterial grouping has encountered significant difficulty centered on the complexity of choosing the correct properties (physicochemical descriptors) upon which to base grouping. It may be likely that the process of grouping depends on the nature of the tests being performed, thus exposure-dependent physical descriptors (EDPDs) should be chosen based on the specific exposure conditions rather than the inherent properties of the nanoparticle in its pristine form. Various grouping approaches have been tested to determine which physicochemical descriptors may be used to establish accurate structure-activity relationships. The use of experimentally derived physical descriptors alone (i.e., size, pH, reactive oxygen species (ROS) concentration, and zeta potential) [11,12], a combination of experimental and theoretical descriptors (i.e., size, electronegativity and molecular orbital energy) [12,13], and solely computational techniques have been explored (i.e., core material, surface coating, and surface charge) [14]. The commonality among these approaches, however, is that they often only consider datasets containing properties relating to the pristine state of nanoparticles, thereby neglecting the impact of biotransformation. Furthermore, the technical barriers for nanomaterial grouping progress relate to data quality and assay selection [15,16]. Specifically, in the case of monitoring silver nanomaterials after ingestion, the lack of standardisation to recreate realistic exposure conditions (in terms of acute and chronic dosing, realistic composition for simulant gastro-intestinal juices, and dynamic digestion protocols) has prevented the identification of suitable descriptors [17,18,19]. Additionally, few studies employ digested nanomaterials to evaluate the cellular response [20,21,22] and characterization of digested materials is sometimes limited to size analysis or dissolution rather than including quantitative characterization of physical descriptors [22,23]. To the best of our knowledge, only one study has shown the speciation of silver nanoparticles in a simulated human oro-gastrointestinal (OGI) tract, highlighting an approximate 75% dissolution of the nanoparticles in the acidic conditions, with the subsequent release of ionic silver, which appears mostly complexed to biomolecules. Although these latter species, also identified in the intestinal tract, are hypothesized to be potentially available for intestinal absorption, no cellular-based studies are present [24]. Hence, despite progress, we continue to lack integrated assays, which relate the biotransformation of nanomaterials in different life cycle contexts to cellular outputs, critical for the generation of EDPDs. Within this context, our study analyzes the full life cycle of ingested silver nanoparticles in the gastrointestinal (GI) tract, including their impact on cellular uptake and integrity using an integrated testing approach coupling acellular and cellular phases. The system integrates two validated testing approaches: a consecutive in vitro gastrointestinal digestion model [25] to obtain a realistic sample of digested nanomaterials, and a well characterized intestinal in vitro model, human colon epithelial cells (Caco-2) cellular monolayer [3,26] to assess cellular uptake and transport across the monolayer. This coupled system is employed to simulate a likely exposure scenario of low and repeated doses exposed to silver nanoparticles over 10 days. Through this approach, a proof of concept for a grouping hypothesis using EDPDs is tested. The behaviour of three test subjects—pristine and undigested AgNPs (undig AgNPs), digested AgNPs (dig AgNPs) and digested silver salts (dig Ag^+^), is analysed and EDPDs (size, crystallinity, chemical core, dissolution, high and low molecular weight silver soluble complexes) are obtained using an interdisciplinary approach. Through the comparison, we determined whether the behaviour of digested particles was akin to the behaviour of non-nano structured silver during simulated oral ingestion. Furthermore, we assessed the grouping hypothesis at the cellular level to verify if cellular responses could be similarly grouped (considering uptake, translocation, membrane integrity, permeability, and inflammation as outcomes).

## 2. Results and Discussion

**Scheme 1:**
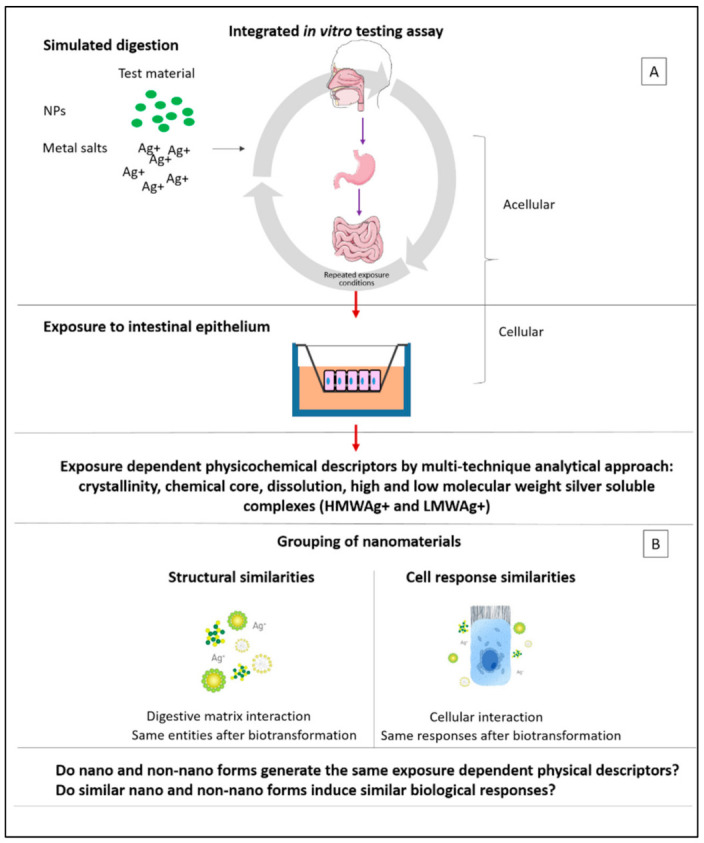
Scheme of the integrated approach used. (**A**) Representation of integrated testing model (acellular and cellular) showing the relationship between each assay step of the experimental process including simulated in vitro digestion of NPs, chronic exposure to Caco-2 intestinal cell monolayers and subsequent analysis. (**B**) Grouping hypothesis employing ‘exposure-dependent physical descriptors’ (EDPDs).

**Integrated in vitro testing model.**Scheme 1A depicts the integrated testing model developed. It links acellular and cellular steps to provide an integrated platform for assessing the behaviour of ingested silver nanomaterials in vitro. As illustrated in the scheme, the initial materials, pristine nanoparticles (AgNPs) and silver salts (Ag^+^) are subjected to a consecutive digestion assay, which simulates the digestive process of NPs through the three digestive compartments (salivary, gastric, and intestinal) (acellular step). The samples generated (dig AgNPs and dig Ag^+^) are subsequently applied to the Caco-2 monolayer using repeated dosing to mimic repeated exposure (cellular step). Undigested nanoparticles (undig AgNPs) are used as negative control (nanoparticles not subjected to digestion that represent an unrealistic exposure scenario). Undigested and digested samples were collected and analysed by an in-depth physicochemical analysis using an interdisciplinary approach (bright field-transmission electron microscope, BF-TEM, scanning transmission electron microscopy–energy-dispersive X-ray spectroscopy ( STEM-EDX), selected area electron diffraction (SAED), ultra-filtration (UF), inductively coupled plasma (ICP)) to quantify the physical descriptors that may be linked to GI biotransformation (from now on referred to as EDPDs throughout the manuscript). Such descriptors are identified in: size, crystallinity, chemical core, dissolution, high and low molecular weight silver soluble complexes (HMWAg^+^ and LMWAg^+^, respectively). They may describe the biotransformation of NPs during the digestion process and demonstrate if nano and non-nano forms of silver are similarly bio transformed to induce similar biological response (i.e., do the nano and non-nano forms generate the same EDPDs? Do similar nano and non-nano forms trigger similar biological responses?) (Scheme 1B).

## 3. Digestion of Silver Nanoparticles (AgNPs) and Ag^+^ by Consecutive In Vitro Digestion Assay (Acellular Phase): ‘What They Are’ in the Gastrointestinal (GI) Tract

**Scheme 2:**
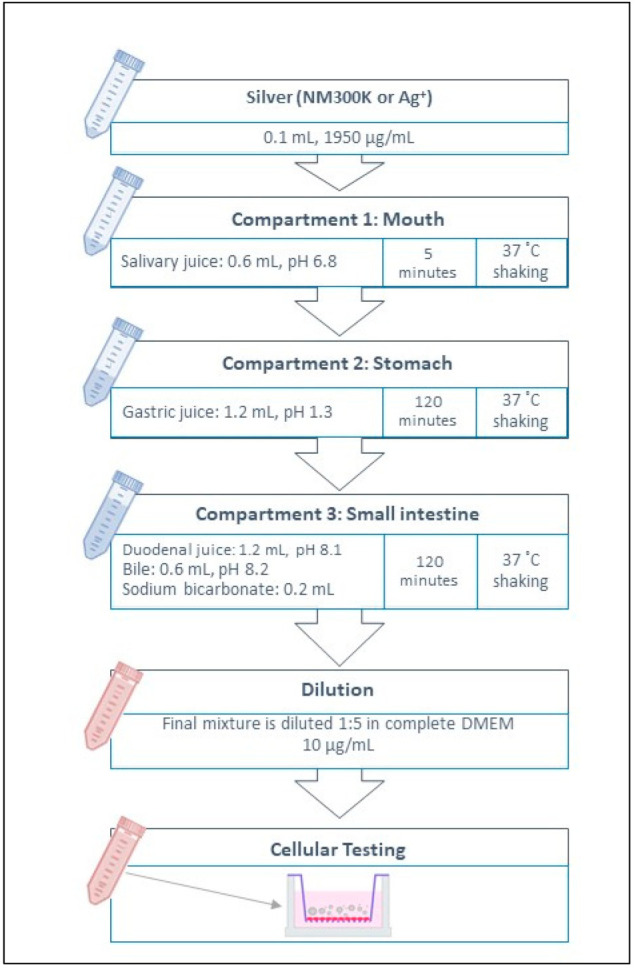
Schematic process for the consecutive in vitro digestive assay showing the conditions required to simulate the passage of material through the mouth, stomach, small intestinal compartments and to cellular testing.

For the present study, silver nanoparticles NM300K were selected from the Joint Research Centre (JRC) Nanomaterials Repository, as a well-studied and extensively characterised reference material. After inclusion in the Testing Programme of the Organisation for Economic Co-operation and Development (OECD) Working Party on Manufactured Nanomaterials (WPMN) and in nanosafety projects [27,28], the particles have undergone physicochemical characterization by several groups independently [29,30,31]. To describe the biotransformation occurring during intestinal digestion (“what they are” during the passage through the GI tract) and extrapolate the EDPDs, pristine AgNPs and Ag^+^ salts were subjected to a modified version of the consecutive in vitro dissolution assay outlined in Bove et al. [25]. As part of a standardized operating procedure (SOP) developed within the Seventh Framework Programme (FP7) European Union (EU) project, “A common European approach to the regulatory testing of nanomaterials” (NANoREG) [32], the selection of this dissolution assay (and, more generally, the decision to perform all experiments using SOPs when available), was pursued to improve adherence for validated methods, thus promoting data reliability for a possible future benchmarking [33]. For the requirements of this study, the protocol was modified in two ways as reported in Scheme 2. Firstly, the starting concentration of digestible material was increased to 1950 µg/mL to produce a resultant solution with a silver concentration of 10 µg/mL. This concentration was selected following a preliminary toxicity screening of NM300K with Caco-2 cells to determine that this concentration was within the sub-toxic range (data not shown). Secondly, the overall volume of the process was scaled down ten-fold to increase its practicality when performed sterilely. The selected concentration was administered to the cells on alternate days for 10 consecutive days and considering a daily assumption of silver by an adult (medium body weight of 70 kg), ideally, the resulted tested mass corresponds to approximately 0.7 µg/kg of bw for the given suspension volume. This dose can be roughly approximated slightly below the current estimated exposure level of silver when employed as a food additive (E174), known to be approximately 2.82–4.78 μg/kg of bw/day for adults [34].

**Figure 1:**
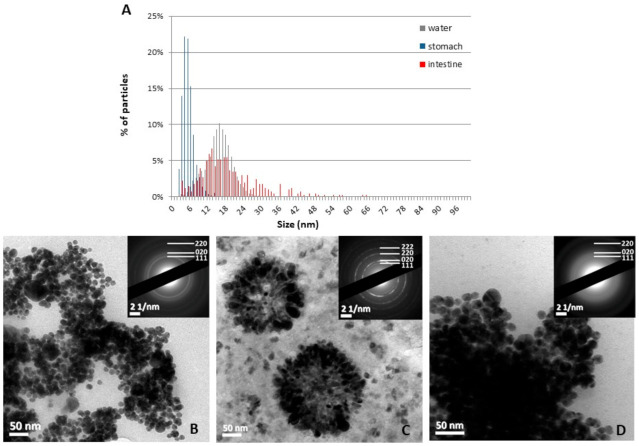
(**A**) Size distribution analysis by transmission electron microscopy (TEM) of undigested silver nanoparticles (Ag NPs) in water (grey); digested in stomach (dark blue); digested in intestine (red) where at least 500 particles for each TEM specimen were analysed by Image J (see method for details). Size: 18 nm ± 5 nm in the water; 5 nm ± 2 nm in the stomach and 17 nm ± 11 nm in the intestine compartments. Bright field TEM images showing silver nanoparticles (AgNPs) (**B**) and AgCl NPs (**C**) found in the stomach after simulated ingestion of AgNPs. Each inset shows the selected area electron diffraction (SAED pattern) acquired from the ROI indicating Ag and AgCl Miller indices (Ag PDF number 01-089-3722 4-783; AgCl PDF number 01-071-5209 31-1238). (**D**) Bright field TEM image and relative SAED pattern acquired from the intestine compartment after simulated ingestion of AgNPs. Consistent with B (Ctrl), the Miller indices correspond to a metallic Ag crystal structure.

To characterize the physicochemical nature of the digested molecular species, the particles were characterised prior to, during and after undergoing the in vitro digestion. The assay was paused at the conclusion of the stomach and intestinal digestive phases where an aliquot sample was removed and characterized by BF-TEM, SAED, High-angle annular dark-field imaging- scanning transmission electron microscopy (HAADF-STEM) and STEM-EDX. Previous data indicate that saliva digestion does not infer specific changes [25]. Hence, as negligible, the biotransformation in the saliva compartment was not considered in this work. The employment of different techniques was key to gain a clear description of the process as a whole, with each technique offering a specialised and complementary perspective. Firstly, statistical size distribution analysis was performed by transmission electron microscopy (TEM) using an SOP developed in NanoREG [35] (Figure 1A,B). Initial size analysis in water of undig AgNPs showed that the particles had ellipsoidal shape with an aspect ratio of 1:1 (major and minor axes of the ellipsoidal sphere reported in Figure S1). The statistical analysis of particles returned major and minor axis mean lengths of 16.5 nm and 13.5 nm respectively with ~97% of particles measuring <25 nm (Figure 1A, grey histograms and Figure S1, grey histograms). These findings are consistent with the NM300K data sheet provided by the JRC, which reports an average size of approximately 15 nm and a narrow size distribution, whereby >99% of particles possessed a size <20 nm. Following the passage through to the gastric compartment, the harshly acidic environment within the stomach leads to significant changes in particle size, whereby >90% of particles measure approximately 5–6 nm (Figure 1A,B and Figure S1, dark blue histograms) and a population of larger structures of size 10–12 nm exist (final tail of the Gaussian curve Figure 1A–C and Figure S1). Moreover, nanoparticles organized in ‘dendritic’ like structures of approximately 200 nm are clearly visible. 

To probe the crystallinity of these nanostructures, we collected SAED patterns from selected regions in Figure 1B–D. The diffraction rings generated from the particles in Figure 1B,D can be assigned to metallic silver, while on the other hand, ‘dendritic’ like nanoparticles, which appear with lower frequency (Figure 1C), generate a pattern ascribable to silver chloride.

**Figure 2:**
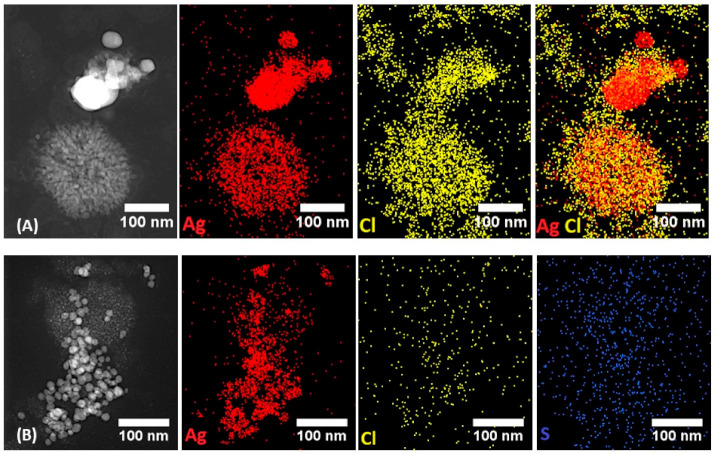
(**A**) Energy-dispersive spectroscopy (EDS) mapping of the stomach digested NPs showing few metallic AgNPs on the upper part of the image (the Cl map confirm, especially in the overlay image, that the Cl in that area probably comes from the stomach matrix but not from the NPs) and a dendritic structure comparable to the one previously shown in the TEM-SAED characterization, which appears to be AgCl; (**B**) EDS mapping of the intestine digested NPs. In this case, no localization of the Cl in the proximity of the NPs has been found. In order to exclude the possible presence of silver sulphide nanocrystals, S map is presented without any co-localization with the NPs.

STEM-EDX analysis (Figure 2A) shows the maps of the most abundant elements (Ag, Cl, S) present in the mixture, which originate from the digestive matrices, as their distribution is not limited to the particles. In particular, in the stomach compartment, few metallic Ag NPs are visible on the upper part of the image (Figure 2A). Moreover, the dendritic structures, larger than those previously shown in the TEM-SAED characterization, agree with the attribution of SAED patterns to AgCl. When the nanoparticles reach the intestinal compartment, the TEM size distribution analysis still shows 5–6 nm nanoparticles (although with a reduced percentage, initial tail of the Gaussian curve in Figure 1A and Figure S1, red histograms) accompanied by populations of nanoparticles ranging from 10 to 60 nm, which are identified as silver nanocrystals by SAED comparable to the Ctrl as reported in Figure 1A (Figure 1A,D and Figure S1, red histograms). However, in the intestinal conditions, by STEM-EDX (Figure 2B), it is difficult to detect AgCl NPs. While known to be poorly soluble [36], it may also be possible that AgCl is present in the stomach, but later transforms into other soluble species or partly precipitates, as also recently evidenced [24]. Importantly, the absence of Ag-S co-localization in the STEM-EDX analysis provides a key insight, suggesting that possible Ag-thiolate species, if present, are more likely to be non-crystalline and thus not detectable by electron diffraction (see X-ray fluorescence (XRF) data below). This latter result is in agreement with recent data by Wu et al. [24]. The capability of Ag^+^ to form soluble complexes in vitro with thiolate biomolecules has been demonstrated, in particular for Cu-binding molecules in which Ag^+^ can replace the native metal [37]. These complexes can form in cellulo as well, and trigger a toxic reaction due to the impairment of the cellular metal homeostasis (for a review, see [36]). In conclusion, the data show an evident dissolution of the pristine nanoparticles into smaller particles in gastric conditions (approximately ≤10 nm) which is in line with previous results [25]. Moreover they also indicate that the dissolution is independent of the starting concentration of NPs which is 10-fold higher here compared with previous studies [25]. Notably, larger nanocrystals incorporating chloride (size range 50–200 nm) are evidenced in the stomach phase. Their formation is assumed to be based on a cooperative mechanism between the agglomeration of partially digested nanoparticles and the concomitant formation of AgCl salts deriving by the released silver ions in Cl-rich media (i.e., the simulant stomach juice applied). This conclusion is supported not only by the crystal nature of the dentritic like structures (Figure 1 and Figure 2), but also by the zeta potential of the resulting nanoparticulates upon biotransformation in the digestive juices (Figure S2). Data indeed show a clear tendency to charge neutralization in the stomach (as opposite to the values of zeta potential obtained in the saliva and intestine compartments, where neutral pHs are applied) and possibly a higher tendency to agglomerate. Our results are in line with outcomes of in vivo and in vitro studies that documented the formation of AgCl precipitates from dissolved AgNPs in Cl-rich media [38]. The toxicity of AgCl has been investigated over different cellular strains, and was found to be comparable to the toxicity of the Ag acetate salt, in which the Ag^+^ ions are bioavailable [38].

In the intestine phase, the dissolution process leads to further biotransformation resulting in newly generated silver nanocrystals with size ranging through 5 to 60 nm, as opposite to undig AgNPs which mainly show dimensions of approximately 20 nm. Ag-S containing nanocrystals (Ag_2_S species) are not detected; however, this does not exclude the presence of soluble non-crystalline Ag-organothiols, as those formed in vivo with glutathione or metallothionein, for instance. 

**Figure 3:**
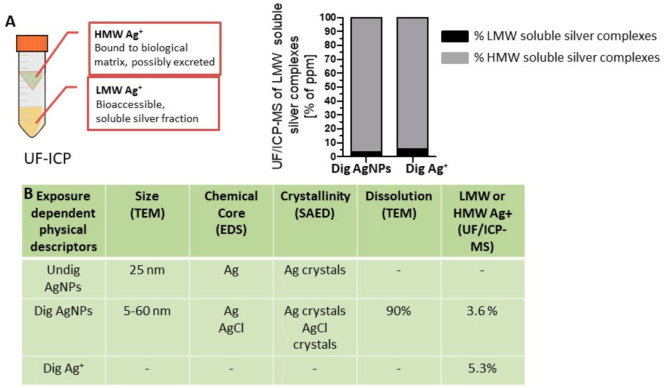
(**A**) Ultra-filtration inductively coupled plasma mass spectrometry (UF-ICP-MS) of high molecular weight (HMW) and low molecular weight (LMW) soluble silver complexes bound to matrix (n = 2, each performed in triplicate); % of LMW soluble silver complexes are: 3.5 ± 0.47% for digAgNPs and 5.38 ± 1.72% for digAg+; (**B**) Comparison of ‘exposure dependent physical descriptors’ among the three silver species highlights similarities and differences between undig AgNPs, dig AgNPs and dig Ag^+^.

Considering the overall size reduction (in diameter) from 20 nm (of undigAgNPs) to 5–10 nm (of digAgNps) in the stomach phase, it may be reasonable to estimate an approximate a 90% reduction in particle volume. This indicates that a considerable portion of silver ions (roughly corresponding to 90% of dissolution) would have been released by the nanoparticles during the digestive process in the stomach phase. However, silver ions possess a very high affinity for Cl^-^ ions and thiolate (R-S) groups of proteins and organic matrices [22,39,40], and therefore we expect that most of the soluble ions released would not remain free. They will bind to the simulated juice components (from now on referred to as “matrix”) composed of proteins and inorganic/organic salts [25] and form different types of soluble silver complexes (throughout the text referred to as ‘soluble silver bound matrix complexes’). They can be reasonably identified as a high MW soluble portion if bonded to proteins, and a low MW fraction if bonded to small chemical molecules such as soluble organic/inorganic salts. To characterize such fractions, UF was applied to perform size-exclusion based separation of the soluble ionic species. When combined with subsequent ICP analysis, UF is an effective technique for quantitative analysis and separation of complex protein mixtures from soluble small molecules, such as drugs or ions [41,42]. Thus, UF-ICP was applied to separate high MW soluble silver-bound matrix complexes, HMWAg^+^ (which are retained on the filter together to the proteins) from low MW ones, LMWAg^+^ (which in turn pass through as eluate). The test was performed in parallel with dig AgNPs and dig Ag^+^ (derived from AgNO_3_ at the equivalent starting concentration of Ag within the AgNPs and corresponding to 100% dissolution) to determine the extent of interaction with the digestive matrix and quantify the amount of soluble fraction (LMWAg^+^). As evidenced by Figure 3A,B, ions from dig Ag^+^ link very strongly with the matrix (95% retention on the filter) leaving only 5.4 ± 1.7% of LMW soluble silver-bound matrix species (LMW Ag^+^). Interestingly, in the case of dig AgNPs where we expect more than 90% dissolution, we found a similar level of LMW Ag^+^ corresponding to 3.6 ± 0.5%. This result shows that, while the vast majority of ions are released by the nanoparticles, they become bound to the digestive matrix as HMWAg^+^ (where they are retained on the filter during UF together with the nanoparticles and proteins of the simulated juices).

To conclude, when taken together, these findings indicate that nanoparticles are biotransformed in the GI tract and cannot be described with the same physicochemical descriptors assigned to pristine nanoparticles to indicate structural similarities (i.e., size, crystallinity, chemical core, dissolved ions and/or soluble complexes) (Figure 3B. Hence, in a grouping hypothesis, it is likely that digested nanoparticles cannot be described by the physical descriptors of pristine nanoparticles nor completely by those employed for salts. This is due to the presence of nanocrystals of differing sizes and core chemistry with respect the undig AgNP (Figure 3B). Concerning the soluble fraction of silver ionic species (which generate by the dissolution) irrespective of its original source (undigested nanoparticles), interaction with the organic/inorganic matrix produces a comparable amount of LMW soluble silver complexes between dig AgNP and digAg^+^ salts (Figure 3B). These complexes are bound to small organic or inorganic salts—likely corresponding to the bioaccessible fraction available for intestinal absorption (approximately 3–5%). Notably, the LMW Ag^+^ descriptors show interesting similarities between dig AgNPs and dig Ag^+^, which will be investigated further during the cellular part of the assay to determine if they correlate to similarities in cellular responses.

## 4. Cellular Response to dig AgNPs and dig Ag^+^ (Cellular Phase): ‘Where They Go and What They Do’ in the Intestinal Epithelium Barrier

Based on the different EDPDs (Figure 3B) whereby nanocrystals with different sizes and chemical nature exist together with a fraction of silver soluble components after digestion, our grouping hypothesis supports the notion that outcomes following the interaction between intestinal cells and undigested nanoparticles or digested nanoparticles could be different due to an intermediate similarity of the latter to dig Ag^+^. 

To verify this hypothesis, we used the cellular model described in Scheme 1. This is a modified version of an SOP developed in the NANoREG project [3], which enables the assessment of nanoparticle transport across a Caco-2 cell barrier. When grown as a confluent monolayer on porous inserts, human colon cells, Caco-2, differentiate, develop characteristics similar to intestinal epithelial cells such as the expression of specific transporter proteins, the formation of microvilli and the development of tight intercellular junctions [43]. To add merit to the protocol, which is most commonly employed to assess the effect of a single dose [44], the cells were maintained in culture for an extended period to allow repeated testing to mimic conditions for realistic nanomaterial exposure [45]. During this time, the cells were exposed to undig AgNPs, dig AgNPs or dig Ag^+^ for 2 h on alternate days for a period of 10 days, to likely mimic repeated ingestion. The biological endpoints chosen to characterise the cellular response were uptake, translocation, barrier integrity, permeability, and inflammation. Each endpoint was measured to verify if the samples tested (undig AgNPs, dig AgNPs and dig Ag^+^) could be grouped based on similarities according to our grouping hypothesis.

**Figure 4:**
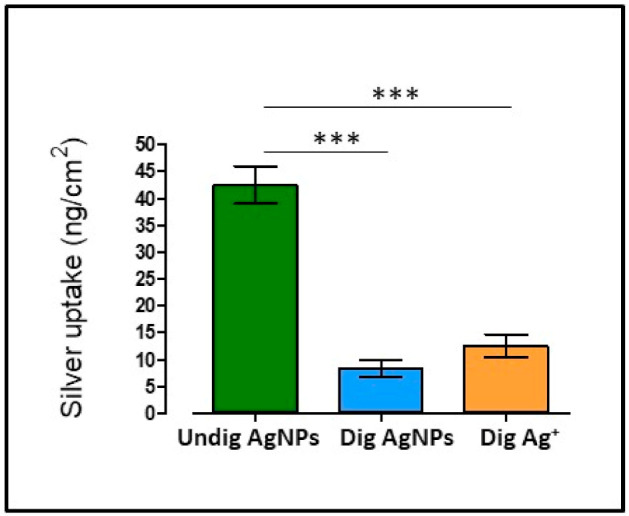
Uptake of undig AgNPs (green), dig AgNPs (light blue) and dig Ag^+^ (orange) by Caco-2 monolayer after a single exposure. Statistical significance is represented with respect to Undig AgNPs (*** *p* < 0.001).

**Uptake and translocation of undig AgNPs, dig AgNPs and dig Ag^+^ silver by intestinal barrier.** The process of uptake and translocation inside and across the intestinal barrier was followed at early time points (i.e., 24 h) by a multi technique approach, which included ICP-MS and micro-beam X-ray fluorescence (µXRF) imaging. This provides corroboration of the individual results by verifying the consistency between bulk and single cell analysis. This combination of techniques and the use of early time points (24 h than end of treatment) allowed us to minimise the uncertainty and analytical artefacts (nanoparticle sticking, aggregation etc.), which often hinder accurate measurements in cellular uptake studies especially in longer treatments [46]. Firstly, we measured the uptake of silver for all samples studied using ICP-MS (Figure 4). Results show that after a single dose at 24 h, the uptake of undig AgNPs is significantly higher (approximately 4-fold) when compared to the uptake of digested species (both dig AgNPs and dig Ag^+^). This suggests that the undigested form may be taken up by cells (and possibly accumulate) with higher efficiency than digAgNPs samples, further confirming the dissimilarity between the two species, as shown by the physicochemical descriptors in Figure 3B. Interestingly, such increased uptake can be explained by the size of EDPD. Cellular uptake of nanoparticles is a process, which is largely dependent on size and concentration [47] with preferential uptake for nanoparticles over corresponding metal salts [48,49]. Moreover, the number of contact sites that the particle’s tangent touches affects particle wrapping and subsequent internalisation with an ideal radial measurement of 15 and 30 nm for cylindrical and spherical particles, respectively, found to optimally induce cellular internalisation [50]. In their undigested form, the quasi-spherical AgNPs are indeed very close to this size range, possibly explaining the observed uptake (which is confirmed also by µXRF, see below). After biotransformation, the wider size range (5 to 60 nm in the intestinal phase) and dissolution leading to the formation of soluble silver species (likely comparable to the LMW silver soluble complexes) and a subsequent reduction in particle number can explain the lower uptake (Figure 4). The translocation study in the basolateral compartment returns values close to the limit of quantification (LOQ) of the instrument for all the species (0.0009 ± 0.00020%, 0.0007 ± 0.00022%, 0.0009 ± 0.00015% with respect to the total dose for undig AgNPs, dig AgNPs and dig Ag^+^, respectively). Although such indications are in line with previous studies for undigested samples [22,39,40,51], here it cannot be informative about similarities among the tested species. 

**Figure 5:**
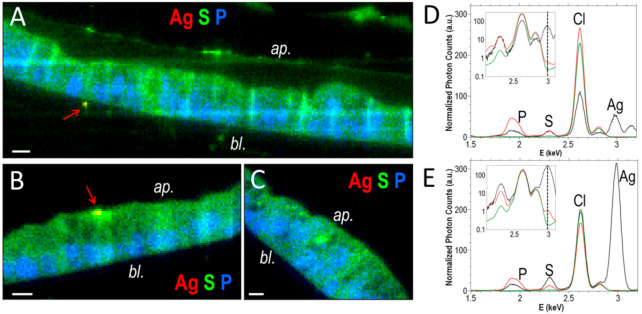
(**A**–**C**) Silver (red), sulfur (green) and phosphorous (blue) distribution in gastrointestinal epithelia exposed to: (**A**) Digested AgNPs, (**B**) undigested AgNPs, and (**C**) digested Ag salt. Elemental distributions were extracted from micro-beam X-ray fluorescence (µXRF) hyperspectral images. Scale bars = 10 µm. The apical (ap.) and basolateral (bl.) sides of the epithelia are indicated over the images. (**D**,**E**) Sum XRF spectra of putative AgNPs (black curves), particle-free cells (red curves) and sample-free resin (green curves) in epithelia exposed to: (**D**) Digested AgNPs, and (**E**) Undigested AgNPs. The areas of interest were selected on maps A and B for digested and undigested AgNPs, respectively, and the sum spectra were normalized by the number of selected pixels. Red arrows in panels A and B point out an intense and localized Ag signal (red appearing yellow due to the co-localization with S, represented in green).

µXRF was applied to investigate the cellular uptake and translocation process of undig AgNPs, dig AgNPs or dig Ag^+^ trying to discriminate between soluble silver complexes and nanoparticulate silver and their relative chemical identification. µXRF images were acquired on sections of gastrointestinal epithelia exposed to undig AgNPs, dig AgNPs or dig Ag^+^. The distribution of phosphorus (P), sulphur (S) and silver (Ag) extracted from hyperspectral images is shown in Figure 5A–C, whereby S distribution (green) highlights the whole cell region, while P (blue) shows a different concentration between the cytosol and nuclei, highlighting the latter. The native element distribution allows the visualization of a monolayer of tightly packed cells. In samples exposed to both undig AgNPs and dig AgNPs, an intense and localized Ag signal (red appearing yellow due to the co-localization with S, represented in green) was detected in selected areas (Figure 5A,B, indicated by red arrows). Interestingly, while the Ag-rich spots are found within the cellular cytoplasm upon exposure to undig AgNPs (Figure 5B), they are detected outside cells following exposure to dig AgNPs (Figure 5A). Moreover, in line with the ICP-MS data indicating a greater uptake for undig AgNPs, the average µXRF spectra of pixels corresponding to putative NPs (Figure 5D,E, black curves) confirm the presence of Ag in such spots, with a much higher concentration (approximately four-fold higher) in the case of exposure to undig AgNPs (Figure 5E) with respect to dig AgNPs (Figure 5D), as indicated by the intensity of the peak at ~3 keV attributed to Ag, which is proportional to the concentration of this element in the selected area. Furthermore, when NPs are detected in cells, as in the case of exposure to undig NPs, their S content revealed by XRF spectra (Figure 5E, black curve) is clearly higher than that of the surrounding cellular environment (red curve), highlighting a local enrichment in S co-localized with Ag. This suggests that, since no pre-digestion is applied, S enrichment around AgNPs stems from interaction with molecular groups, most probably thiols, occurring *in cellulo*. Although dig NPs are detected outside cells, (i.e., in the embedding resin) the sulfur content in the NPs (Figure 5D, black curve) is higher than in the resin (green curve), and rather comparable to cellular S content (red curve). These findings, as also postulated by the EDX data, suggest that S enrichment around dig AgNPs originates from the digestion matrix forming non-crystalline organothiol complexes, since their absence in EDS analysis rules out the possibility that crystalline AgS forms exist (Figure 2). This result is in agreement with recent findings showing Ag-S species including either insoluble Ag_2_S (silver sulfide) precipitates [52,53] or soluble non-crystalline Ag-organothiol complexes, which form upon AgNP biontransformation in GI tract [25,54,55]. It is worth noting that the high concentration of Cl in the embedding resin (Figure 5D,E green curves), expected considering that the general formula of epoxy resins is C_21_H_25_ClO_5_, does not allow clear conclusions to be drawn on the presence of Cl in Ag-rich areas by µXRF.

**Figure 6:**
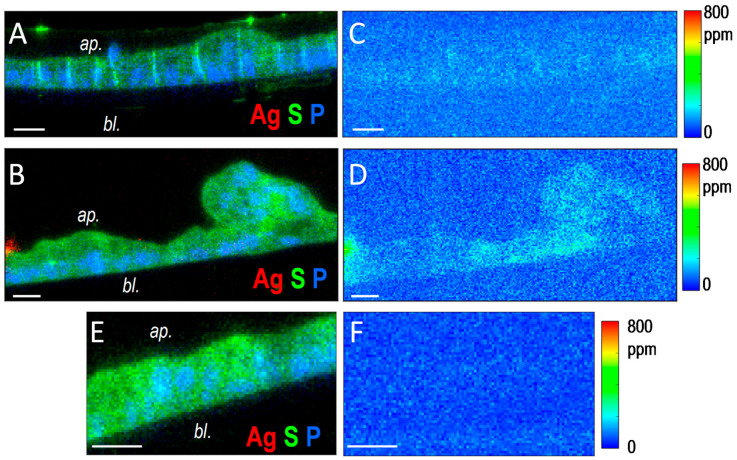
XRF: silver (red), sulfur (green) and phosphorous (blue) distribution in gastrointestinal epithelia exposed to: (**A**) dig or (**B**) undig AgNPs, and (**E**) in untreated cells. The apical (ap.) and basolateral (bl.) sides of the epithelia are indicated over the images. False-color representation of Ag concentration in the same areas of gastrointestinal (GI) epithelia exposed to: (**C**) dig AgNPs or (**D**) undig AgNPs, and (**F**) in untreated cells. Elemental distributions and concentrations were extracted from µXRF hyperspectral images. Scale bars = 20 µm.

Particle-free areas of NP exposed samples are considered in order to be able to analyse also soluble silver matrix-bound complexes (Figure 6). Here, a low and diffused Ag signal is clearly visible for undig AgNPs (Figure 6D). The corresponding Ag concentration per pixel is estimated in the 100–200 ppm range, consistent with the value measured previously in particle-free cells under the same exposure condition (Figure 5B,E). These data are in line with current literature showing a similar signal by XRF in HepG2 cells exposed to AgNPs, which were attributed to the release of Ag ions upon lysosomal dissolution of the NPs *in cellulo*, following combined TEM and X-ray Absorption Spectroscopy (XAS) analysis [56]. This supports findings in other cell lines for pristine AgNPs with different surface coatings: NPs enter the cell by endocytosis and in part dissolve in the acidic environment inside the vesicles. This behaviour results in liberation of Ag^+^ ions into the cellular cytoplasm, which subsequently bind to thiolate groups of proteins and biomolecules [56,57]. Notably, this diffused Ag signal is absent in cells treated with dig AgNPs or is indistinguishable from the background level (Figure 6C compared with untreated cells in Figure 6F). A similar scenario is encountered when cells are exposed to dig Ag^+^: no diffused Ag signal is apparently detected by XRF hyperspectral images, as suggested by the absence of a red signal associated to Ag in Figure 5C. When the XRF spectra relative to the whole cell region is averaged (Figure S3), the absence of a peak at the characteristic XRF emission energy of Ag confirms this observation. According to ICP measurements (Figure 4), the cellular amount of Ag in epithelia exposed to dig AgNPs and dig Ag^+^ is four times lower than in the case of exposure to undig AgNPs. Considering this, and that Ag concentration in particle-free areas of cells exposed to undig AgNPs is barely distinguishable from the background (Figure 5E, inset, red curve), it is reasonable to think that also dig AgNPs and dig Ag^+^ samples dissolve in cellulo; however, these values cannot be detected by our experimental conditions being lower than the detection limit. A more detailed interpretation of the XRF spectra of Figure 5E is reported in the supporting information (SI). Taken together, these findings support the grouping hypothesis, demonstrating that dig AgNPs and dig Ag^+^ can be grouped based on the similarity of a comparable amount of non-particulate Ag, which is most likely soluble silver matrix-bound complexes which occur during digestion (Figure 3, Figure 5 and Figure 6). On the side of nanoparticulates, dig AgNPs behave differently to undig AgNPs due to extensive dissolution in GI tract and subsequent biotransformation in less amount of Ag and AgCl nanocrystals (Figure 1 and Figure 2). This can also corroborate the lesser extent of cellular uptake (Figure 4). Finally, as for the ICP-MS data, by µXRF, a diffused Ag signal within the basolateral compartment, if present, is below the detection limit and does not allow discrimination between translocated matrix-bound and free soluble complexes.

**Figure 7:**
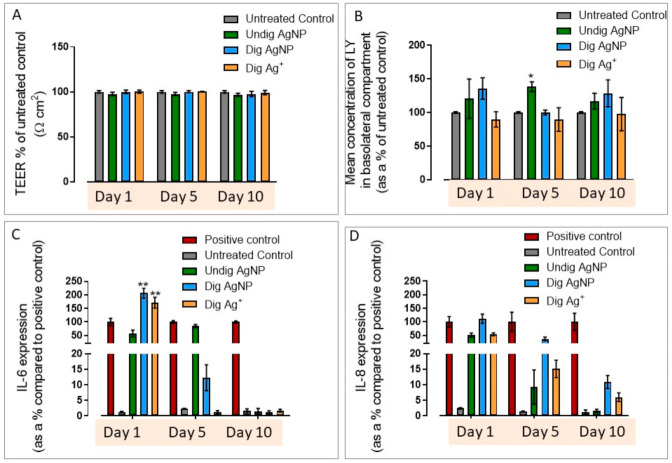
(**A**) Trans-epithelial electrical resistance (TEER) measurements of cell monolayers and (**B**) Lucifer yellow (LY) permeability assay results for undig AgNPs (green), dig AgNPs (blue) and dig Ag^+^ (orange) as compared with untreated control cell layers (grey) on days 1, 5 and 10. Statistical significance is represented respect to untreated control (* *p* < 0.05). (**C**) interleukin-6 (IL-6) and (**D**) IL-8 release in the apical medium following chronic exposure to the three silver species on days 1, 5 and 9 presented as a percentage compared to the response generated by the positive control (composed of 25 ng/mL IL-1β, 1 µg/mL lipopolysaccharide (LPS), 50 ng/mL tumor necrosis factor apha (TNFα) and 50 ng/mL interferon gamma (IFN-γ)). Statistical significance is represented with respect to positive control (** *p* < 0.01).

**Intestinal barrier integrity, permeability and inflammation.** The integrity of the intestinal layer upon chronic incubation with the three silver samples was assessed by measuring the trans-epithelial electrical resistance (TEER) and the passage of Lucifer yellow (LY), (a marker of paracellular transport) into the basolateral (Bl) compartment (Figure 7A,B). TEM microscopy analysis was also employed for the same purpose, and in line with µXRF, highlights a tightly packed and uniform barrier with clear presence of unaltered tight junctions, microvilli and desmosomes (representative images for all treated cells are reported in Figure S4). Epithelial layers treated with undig AgNP, dig AgNP and dig Ag^+^ showed neither alterations of these cellular structures nor detectable differences in TEER results with respect to untreated controls at any time of exposure (Figure 7A).

Regarding the LY data, instead, while at day 5 a statistically significant change in LY passage (Figure 7B) was shown by cells treated with undig AgNPs, later the effect is recovered. These results indicate that the biotransformed digested samples induced no disruption or perturbation of the intestinal barriers after repeated exposure, while the undig AgNPs presented a transient alteration mid-way through the treatment period (Figure 7B). This effect, although weak and transient, evidences a different response of the undig AgNPs, with respect the digested samples previously grouped together by the similarity in term of soluble silver fraction.

In vitro and in vitro studies indicate that inflammation is a common outcome following ingestion of metal nanoparticles [58,59,60,61]. Inflammation can be due to the intracellular release of metal ions occurring via lysosomal degradation after nanoparticle endocytosis causing cell toxicity [27,62,63]. Therefore, the release of proinflammatory cytokines, linked to acute (interleukin-6 (IL-6) and IL-8) and chronic inflammation (IL-6) [64], was measured in the apical and basolateral media to evaluate if chronic exposure to the three silver species triggers an inflammatory response in Caco-2 cell monolayers. No cytokines were detected in the basolateral fluids (data not shown), suggesting a polarized secretion of cytokines by epithelial cells into the apical compartment, as also reported by other studies for human epithelial cell lines when grown on Transwell^TM^ inserts [65,66]. After 24 h, exposure to undig AgNPs, dig AgNPs and dig Ag^+^ produces an increase of IL-6, that becomes statistically significant for the digested species: approximately two-fold higher than the response generated by the positive control (Figure 7C). At the same time point, this response was less pronounced in the case of undig AgNPs although higher than the negative control, providing evidence that the kinetics that the undigested and digested species follow to induce an inflammation response are different. This response has the characteristics of an acute effect with the cells showing complete recovery after 10 days for all treatments (Figure 7C) [67]. However, also in the recovery phase, cells seem to react differently to the digested species showing first a higher inflammation response, but also a more rapid kinetics to recover a physiological status. A similar decrease in IL-6 level has been observed by other groups over short time periods while continued testing revealed a resurgence in IL-6 levels at longer times [68]. In these experimental conditions, our data support the profile of IL-6 as an acute, rapidly secreted but transient cytokine that is upregulated at the onset of the injury or infection and known to be responsible for symptoms such as fever [69]. In parallel, for IL-8, we observed a higher secretion after cell exposure to all treatments with respect to the negative control, but lower or not statistically significant compared to the positive control (Figure 7D). However, the kinetics that the cells treated with the digested species follow to recover normal conditions appear again to be quite different from those treated with undigAgNPs. It is likely that the cells react more efficiently to the inflammatory stimulus induced by the digested species, but, similarly rapid, are able to recover it.

The overarching message of these results indicate a similarity in the behaviour of digested samples (both digAgNPs and dig Ag^+^). Specifically, the data indicates no alteration of intestinal barrier integrity and a weak inflammatory response, which is completely recovered after 10 days. While these effects are observed following the experimental conditions applied within this study, the possibility that doses of increased concentrations or frequency (potentially occurring in a chronic daily assumption of silver nanoparticles) generating a more severe (and less transient) effect cannot be excluded.

## 5. Conclusions

**Scheme 3:**
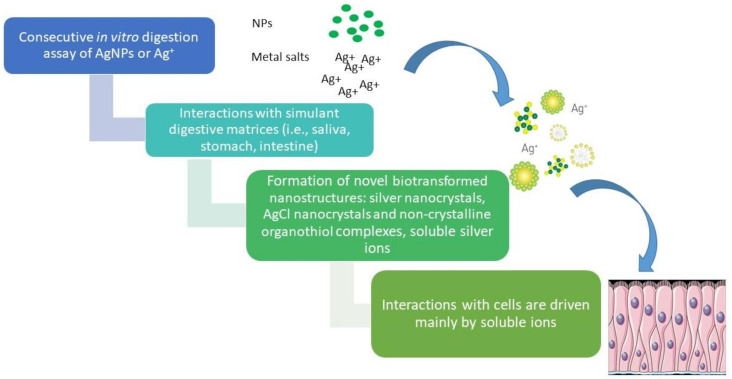
Schematic representation of the biotransformation by consecutive in vitro digestion assay and generation of soluble silver ionic complexes which trigger a cellular response of the intestinal epithelium.

The expanding use of new nano-enabled products demands innovation in the area of nanomaterial risk assessment. The need for materials grouping based on structural similarities is reliant on the ability to identify appropriate physical chemical descriptors quantified in real exposure scenarios. Our work, using silver nanoparticles as a case study, demonstrates a proof of concept that highly or partially dissolved silver nanoparticles can behave similarly to the corresponding saline form. Similarity is identified in terms of interaction of soluble released silver complexes with biological matrices and the ensuing cellular responses. Our study highlights that undigested nanoparticles are unrealistic samples when assessing their effect following human ingestion. Indeed, the EDPDs identified upon in vitro digestion (size, crystallinity, chemistry of core material and LMW silver soluble complexes which derive by dissolution) do not allow us to identify dig AgNPs similar to the undigested form. Furthermore, the presence of similar amounts of the soluble silver ionic components corroborates a similarity between dig AgNPs and dig Ag^+^. For this reason, we propose that the similarities observed in the two digested samples for uptake and inflammation are possibly driven by the soluble components present in the two samples. These findings and the role of soluble silver ionic complexes to trigger a cellular response are summarized in the Scheme 3 underlining the importance of testing materials within realistic exposure conditions to avoid creating misleading results, derived from an implausible scenario. Therefore, to accurately determine the effect of nanoparticulate silver on the human intestinal layer, we propose the integration of an acellular in vitro test to precede cellular analysis. We foresee that this type of integrated analysis may help to identify accurate ‘exposure based physical descriptors’ which are critical for interpretation of biological responses and subsequent grouping.

## 6. Materials and Methods

**Chemicals and Reagents.** All chemicals and reagents used were obtained from Sigma-Aldrich (Milan, Italy), unless otherwise stated.

**Preparation of silver materials.** Silver nanoparticles NM300k were obtained from the repository list of the European Commission Joint Research Centre (JRC). The NM300k stock solution was diluted to 2.56 mg/mL, adhering to the NANOGENOTOX dispersion protocol for nanomaterials [70]. Following dispersion, a further dilution to 1950 µg/mL was performed to prepare the material for the in vitro dissolution assay. Following digestion, the suspension of digested AgNPs (Dig AgNPs) was diluted five-fold (final concentration 10 µg/mL) in cell culture medium to prevent cellular toxicity, which can result following contact with concentrated gastrointestinal juices. For undig AgNPs, following the application of the dispersion protocol, NM300k was diluted to 50 µg/mL before a further five-fold dilution in cell culture medium. AgNO_3_ was purchased from Sigma Aldrich (S8157) and was used to prepare a 1950 µg/mL solution to use as the starting material for the in vitro dissolution assay. Following digestion, the solution was diluted five-fold in cell culture medium prior to use (dig Ag^+^). In all cases, the same final concentration (FC) for all tested silver materials was used and corresponded to 10 µg/mL.

**In vitro dissolution assay.** To simulate digestion, a modified version of the in vitro dissolution test described by Bove et al. [25] was employed. This assay is validated as an SOP within an EU project (NANoREG D2.08 SOP 06) [28] and it is currently under validation through the OECD Working Party on Manufactured Nanomaterials (WPMN) (ENV/CHEM/NANO (2019)5/ADD1. The physiological relevance of the method is given by the composition of the simulant fluids (components typical of human saliva, stomach and intestine fluids) and by the cascade addition of digestive juices to simulate the dynamic changes of the molecular compositions, the relative concentrations and of the pH jumps occurring within the digestive environments that foods or nanoparticles can encounter during the transit through the oro-gastrointestinal tract. Moreover, the incubation times selected for the test (5 min of saliva, 2 h of stomach and 2 h of intestine) derived from published information which refer to the digestive times of stomach and intestine emptying in humans in fasted conditions [71,72,73]. To prevent the contamination of cell cultures the digestive juices were prepared in a sterile environment, and the digestive assay performed aseptically. While sterility can be achieved by filtering solutions with a 0.22 μm filter, in the case digestive juices containing mucin, pancreatin and lipase, their precipitation caused filter blockage. For this reason, an alternative method of sterilisation—exposure to UV light for 30 min, was employed to achieve sterility of these components. This treatment has previously been shown to have no effect on the enzymatic activity of such compounds [74]. All digestive juices were prepared on the day prior to their use, and incubated overnight at room temperature. The juices were then heated to 37 °C for 2 h before use. Full details of the composition of the juices can be found in the supporting information (Tables S6–S9) while briefly, the properties of the various mixtures can be described as follows: (a) simulated saliva was composed of ions (ions derived from sodium, potassium and calcium salts), organic compounds and proteins (pH 6.8 ± 0.1); (b) simulated gastric juice was composed of ions (ions derived from sodium, potassium and calcium salts), carbohydrates, urea and proteins (pH at 1.3 ± 0.1) and (c) simulated small intestine juice was made up of a combination of duodenal juice (pH 8.1 ± 0.1) bile (pH 8.2 ± 0.1) and sodium bicarbonate. To begin with, 0.1 mL of 1950 µg/mL silver (AgNPs or Ag^+^) was transferred into a 15 mL falcon tube. The digestion commenced when 0.6 mL of saliva juice was added to the tube. Following the addition, the tube was sealed, inverted on a 45° angle, and placed inside an orbital incubator shaker and held at 37 °C, 120 RPM for 5 min in the light. Subsequently, 1.2 mL of gastric juice was added before the tube was replaced in the incubator for 120 min. Finally, 1.2 mL of duodenal fluid, 0.6 mL of bile salts and 0.2 mL of 84.7 g L^−1^ sodium bicarbonate were added before a final incubation for 120 min. All additions were performed aseptically inside a laminar flow hood. Upon the conclusion of the digestion process, the resulting solution represents a realistic biotransformed test material with a final silver concentration of 50 µg/mL.

**Cell culture.** Human colon epithelial (Caco-2) cells were kindly provided by Dr. Isabella De Angelis, Istituto Superiore di Sanità (ISS), Rome, Italy) and were obtained from ATCC Cell lines bank. Dulbecco’s modified Eagle medium (DMEM) culture medium, TrypLE Select, non-essential amino acids (NEAA), penicillin (10,000 U/mL), streptomycin (10,000 µg/mL) and Hank’s Balanced Salt Solution (HBSS) buffer were purchased from Invitrogen (Milan, Italy). Phosphate-buffered saline (PBS) without calcium and magnesium was purchased from Lonza (Milan, Italy), and fetal bovine serum (FBS) from HyCLone (Milan, Italy). Culture flasks and Transwell^®^ inserts were purchased from Corning (Milan, Italy) and Merck (Milan, Italy) respectively. Caco-2 cells were maintained in a 25 cm^2^ culture flask at 37 °C in humidified atmosphere with 5% CO_2_. Caco-2 cells were grown in complete DMEM, prepared by supplementing DMEM culture medium with 10% (final) FBS, 1% (final) NEAA and 1% (final) PenStrep. Cells were maintained in a 25 cm^2^ culture flask at 37 °C in humidified atmosphere with 5% CO_2_ until reaching ~80% confluence. Confluent cells were sub-cultured and the culture medium was changed three times per week. Complete DMEM was prepared by supplementing DMEM culture medium with 10% (final) FBS, 1% (final) NEAA and 1% (final) PenStrep. Cells were sub-cultured upon reaching ~80% confluence, changing the culture medium three times per week.

**Nanoparticle permeability assay.** Following the NANoREG SOP “Standard Operating Procedure for evaluation of NPs impact on Caco-2 cell barrier model” [75], Caco-2 cells were seeded at 1.7 × 10^5^ cells/cm^2^ apically on Transwell permeable cell culture inserts (Corning, Milan, Italy) (pore size 1μm) housed within 12 well plates and maintained for 21 days to allow full differentiation. Apical and basolateral media were changed every 3 days. Once ready, cell inserts were then incubated with dig AgNPs or dig Ag^+^ diluted 1:5 in cell culture medium, or undig AgNPs at the final concentration of 10 μg/mL for 2 h every 2nd day in order to mimic chronic silver exposure. As a control, cell inserts were first incubated with digestive juices alone (diluted 1:5 in cell culture medium) to verify that this had no effect to cell viability. All treatments were performed in triplicate. Prior to commencing treatment, the integrity of the tight intracellular junctions were assessed by measuring the TEER of each layer using a “chopstick electrode” connected to a Millicell ERS voltohmmeter (Millipore, Milan, Italy). Only cell layers which returned a TEER value > 200 Ω.cm^2^ were used. TEER measurements were repeated on Day 1, 5 and 9 for comparative assessment maintaining the cells at 37 °C on a temperature-controlled heating plate. TEER values are presented as a percentage of control determined from untreated cellular layers maintained in identical conditions. Values (expressed as Ohms (Ω) × cm^2^) are shown as the mean of triplicate measurements, with a blank (cell-free insert) resistance value subtracted according to the following equation:

TEER = [Ω cell monolayer − Ω filter (cell-free)] × filter area (1.12 cm^2^)

The impact of silver treatment on epithelial integrity was also evaluated using the Lucifer yellow (Sigma-Aldrich, Milan, Italy) assay. The ability of the marker to passively cross the monolayer via the paracellular space is an additional method to assess changes to the tight junctions in which can occur in response to stress. The LY assay was performed on days 1, 5 and 9. Cell inserts and basolateral compartments were washed with HBSS and treated with LY (0.4 mg/mL) apically, before being incubated for 120 min. Basolateral samples were collected in duplicate from all wells and transferred to a black 96-well plate for fluorometric measurement (ex. 428 nm, em. 536 nm) to determine the LY content. The transport is represented as the percentage of LY passage into the basolateral side following treatment compared to the percentage of LY passage through untreated control layers.

**Cytokine analysis.** The amount of human interleukin-6 (IL-6) and human interleukin-8 (IL-8) were measured in both apical and basolateral side of the cell cultures. Human IL-6 enzyme-linked immunosorbent assay (ELISA) MAX™ Deluxe and Human IL-8 ELISA MAX™ Deluxe (Campoverde, Italy) were used. Assays were carried out following the manufacturers’ protocols. The optical density of each well was determined at 450 nm and corrected by subtracting the optical aberration of the 96-well plastic plate at 570 nm. As nanoparticles could interfere with the ELISA assay, the IL-6 and IL-8 standards were dissolved in the assay diluent (as for manufacturer’s protocol) or in assay diluent spiked with silver NPs tested. We found that the silver NPs tested affected the ELISA readouts, quenching the absorbance signal, as shown in Figure S5. Hence, cytokine concentrations in supernatants were extrapolated using the ELISA calibration curves of each silver NPs. A mix of IL-1beta (25 ng/mL), lipopolysaccharides (LPS) (1 µg/mL), tumor necrosis factor alpha (TNFα) (50 ng/mL) and interferon gamma (IFN-γ) (50 ng/mL) was used as positive control (24 h of incubation) [76].

**Zeta-potential analysis of digested silver NPs in simulant digestive juices.** The zeta potential of the resulting nanoparticulates upon biotransformation in the digestive juices was followed and measurements were taken after the respective incubation times in the simulant digestive juices (namely 5 min, 120 min and 120 min, respectively after incubation in the simulant juices as they are obtained by the cascade addition). Ctrl is reported in the table and represents the surface charge of NPs dissolved in ultrapure water. Each suspension (47.5 µg/mL), at the end of the incubation time, was centrifuged at 20,000 g for 15 min at 4 °C and corresponding pellet was washed three times with a volume of Milli-Q^®^ water equal to the initial volume [77]. The surface charge of the resulting pellets and Ctrl was measured via Zeta Potential (ZetaP) analysis using a Zetasizer Nano-ZS (Malvern, UK) at 25 °C. Three consecutive measurements were run for each sample.

**Inductively coupled plasma mass spectroscopy (ICP-MS).** Elemental analysis was carried out by inductively coupled plasma mass spectroscopy (ICP-MS,model iCAP-TQ (Thermofisher, Milan, Italy)) following EPA Method 6020B [78] on basolateral medium and cellular layers collected on day 1 (24 h after the first treatment) to determine cellular uptake and translocation of silver species. Medium collection was performed using a micropipette, leaving the cellular layer inside the Transwell filter (Corning, Milan, Italy) where it was washed thoroughly to remove nanoparticles adhered to the surface. Samples were collected in 10 mL Pyrex pressure tubes (CEM) (Corning, Milan, Italy) and treated with 70% nitric acid before being digested by microwave using a Discovery SPD (CEM) system (Corning, Milan, Italy) prior the analysis.

**Ultrafiltration (UF).** Ultrafiltration of samples was performed using Amicon Ultra 0.5 mL centrifugal 3 K filters (Merck Millipore, Milan, Italy). To perform UF, 0.5 mL of digested sample was loaded into the filter unit before being centrifuged at 14,000× *g* at room temperature for 30 min. The samples obtained were extensively washed with water (3 cycles at least).

## 7. Transmission Electron Microscopy (TEM)

*Size distribution analysis by TEM.* The size distribution analysis of dig AgNPs and morphology analysis in simulated GI tract juices were performed by TEM (JEOL JEM 1011 microscope (JEOL, Tokyo, Japan) working at 100 kV accelerating voltage). TEM samples were prepared and analyzed following an SOP developed in the EU NANoREG project [30] and as reported in Bove et al. [23]. Briefly, TEM samples were prepared according to the grid on drop method. 10 μL of the diluted dispersion was pre-treated with 1% Alcian blue (Sigma-Aldrich, Milan, Italy) and put in contact with a carbon-coated, 400 mesh carbon grids (Agar Scientific, Essex, UK). After 10 min of contact with the sample, the grid was blotted to remove the excess of sample and left to air-dry at room temperature. To obtain an estimation of the repeatability (within one-day variability) and intermediate precision (day-to-day variability), three (different) vials of the samples were analysed on three days within one week; on each day, two TEM specimens were prepared from each vial. Micrographs of ten regions and at least 500 particles of every TEM specimen were acquired randomly at the magnification, pixel size 0.56 nm, and micrograph size 1511.61 × 1500.36 (nm) (not reported in the manuscript). Micrographs were then analysed by the Image J software (National Institutes of Health, Wisconsin, WI, USA) performing a manual grey-scale thresholding and binarization. Afterwards, to separate touching particles, a separate particle filter was applied (particles touching the bottom and right and left sides of the image were rejected). At the end of the image analysis, particles with circularity between 0.5–1 were considered as single primary particles. A representative micrograph and corresponding annotated micrograph were reported for each NM300k specimens.

*Characterization of NM300K during digestion.* Brightfield images and selected area electron diffraction (SAED) analysis of the AgNPs at different digestion stages were obtained by a JEOL JEM 1011 (JEOL, Tokyo, Japan), working at 100 kV accelerating voltage. High-angle annular dark field-scanning TEM (HAADF-STEM) images were acquired using a JEOL JEM-2200FS microscope (JEOL, Tokyo, Japan) equipped with a Schottky emitter operated at 200 kV and a CEOS spherical aberration corrector of the objective lens (all the equipment was obtained from JEOL, Tokyo, Japan). Energy-dispersive X-ray (STEM-EDX) analysis of the Ag nanoparticles previously diluted in the cell culture medium were obtained in STEM mode on the same microscope equipped with a Bruker Quantax 400 system with a 60 mm^2^ silicon-drift detector (SDD).

*Caco-2 barrier microscopy analysis.* The Caco-2 cellular barriers were fixed with 1.5% glutaraldehyde in 0.1 m sodium cacodylate buffer (pH 7.4) for 2 h, post fixed with 1% osmium tetroxide in the same buffer and stained overnight with 1% uranyl acetate. Then, the samples were dehydrated with a graded ethanol series. Dehydrated samples were infiltrated with propylene oxide, before embedding in epoxy resin (Epon 812, TAAB). Sections of the embedded cell barriers were obtained by an ultramicrotome (UC6, Leica, Milan, Italy) equipped with a diamond knife (Diatome). Images were acquired with a JEOL JEM 1011 (JEOL, Tokyo, Japan) electron microscope, working at a 100 kV acceleration voltage, and equipped with a Charge-Coupled Device (CCD) camera (Gatan Orius SC-1000, Pleasanton, CA, USA).

**Micro beam X-ray fluorescence (µXRF) analysis.** Sample preparation for XRF imaging followed the protocol of fixation and inclusion in resin already described for TEM, with the only difference that OsO_4_ post-fixation and uranyl acetate staining were avoided. Sections 1 µm thick were cut and sandwiched between two Ultralen^®^ foils (Leica, Milan, Italy) for XRF analysis. XRF hyperspectral images were acquired on the beamline ID21 [79] of the European Synchrotron Radiation Facility (ESRF, Grenoble, France), at room temperature and under high vacuum (10^−5^ mbar). The photon beam energy was tuned to 3.5 keV by using a fixed exit double-crystal Si(111) monochromator (ΔE/E ~ 10^−4^). The X-ray beam was focused to 1.3 × 0.5 µm^2^ (H × V) with a Ni-coated Kirkpatrick–Baez mirror system (designed at ESRF for the ID21 microscope, based on Ni coated mirrors from Zeiss, Germany), yielding a flux at sample of 1.3·10^10^ photons/s. The fluorescence emission signal from the sample was detected with an energy-dispersive, large area (80 mm^2^) SDD detector equipped with Be window (SGX from RaySpec Ltd, High Wycombe, UK). Images were acquired by raster-scanning the sample in the X-ray focal plane, with a 1 × 1 µm^2^ step and 500 ms dwell time. Ag was detected through a L_α1_ emission line at 2.984 keV. The detector response was calibrated over a thin film reference sample consisting of layers of elements in ng/mm^2^ concentration sputtered on a 200 nm thick Si_3_N_4_ membrane (RF7-200-S2371 from AXO, Dresden GmbH, Dresden, Germany). XRF images were normalized by the incoming flux and subjected to spectral deconvolution with the PyMca software package version 5.4.2 developed at ESRF, Grenoble, France [80]. Elemental mass fractions were calculated from fundamental parameters, assuming as matrix the soft tissue composition provided by the National Istitute of Standards and Technology (NIST) Star database (https://physics.nist.gov/cgi-bin/Star/compos.pl?matno=261, accessed on 8 June 2018), with a density ρ = 1.0 g/cm^3^. Two to five XRF hyperspectral images per condition (including control) were measured and processed, over at least two different sections, providing consistent results.

**Statistics**. All results are presented as a mean ± standard deviation. One-way analysis of variance (ANOVA) was applied to determine statistical significance between groups. Results were considered statistically significant, if *p* < 0.05 denoted by *.

## Data Availability

Not applicable.

## Supplementary Materials

The following are available online at https://www.mdpi.com/article/10.3390/nano11061587/s1, Figure S1: TEM Size distribution analysis of NM300K. Figure S2. Zeta potential of digested AgNPs by consecutive in vitro digestion assay. Figure S3: XRF analysis of gastrointestinal epithelia exposed to Ag salts. Figure S4: TEM image of Caco2-monolayer upon treatement with 10 ug/mL of undigAgNPs, digAgNPs, and dig Ag+; Figure S5: Interference evaluation of the NPs with the enzyme-linked immunosorbent assay (ELISA). Table S6: Molecular composition of the saliva juice. The reported values are related to the final concentration used in the digestion assay, Table S7: Molecular composition of the stomach juice. The reported values are related to the final concentration used in the digestion assay, Table S8: Molecular composition of the duodenum juice. The reported values are related to the final concentration used in the digestion assay, Table S9: Molecular composition of the bile juice. The reported values are related to the final concentration used in the digestion assay.

## Abbreviations

AgNP	Silver nanoparticle
EDPDs	Exposure dependent physical descriptors
Bl	Basolateral
BFTEM	Bright field transmission electron microscopy
Dig Ag^+^	Digested silver salts
Dig AgNP	Digested silver nanoparticle
Undig AgNP	Undigested silver nanoparticle
HMWAg^+^	High molecular weight silver soluble complexes
LMWAg^+^	Low molecular weight silver soluble complexes
EU	European Union
OGI	Oro-Gastrointestinal
GI	Gastrointestinal
HAADF	High-angle annular dark-field
ICP-MS	Inductively coupled plasma mass spectrometry
IL	Interleukin
LPS	Lipopolysaccharide
JRC	Joint Research Centre
LY	Lucifer yellow
MW	Molecular weight
NP	Nanoparticle
OECD	Organization for Economic Co-operation and Development
ROS	Reactive oxygen species
SAED	Selected area electron diffraction
SOP	Standard operating procedure
STEM	Scanning transmission electron microscope/y
EDX	Energy dispersive X-ray spectroscopy
TEER	Transepithelial electrical resistance
UF	Ultrafiltration
µXRF	Micro X-Ray Fluorescence
WPMN	Working Party on Manufactured Nanomaterials
XAS	X-ray absorption spectroscopy
