# Supplementary material for: Biotransformation of Silver Nanoparticles into Oro-Gastrointestinal Tract by Integrated In Vitro Testing Assay: Generation of Exposure-Dependent Physical Descriptors for Nanomaterial Grouping
Source: Nanomaterials (Basel). 2021 Jun 17;11(6):1587. doi: 10.3390/nano11061587 (PMC8233905; doi:10.3390/nano11061587)
Supplement: Supplementary file 1 [file nanomaterials-11-01587-s001.zip › nanomaterials-1219106-supplementary.pdf]

Supporting Information

Biotransformation of Silver Nanoparticles into Oro-Gastrointestinal Tract by Integrated In vitro Testing Assay: Generation of Exposure-Dependent Physical Descriptors for Nanomaterial Grouping

Catherine Carnovale [1,†], Daniela Guarnieri [2,3,†], Luisana Di Cristo [1], Isabella De Angelis [4], Giulia Veronesi [5,6], Alice Scarpellini [7], Maria Ada Malvindi [8], Flavia Barone [4], Pier Paolo Pompa [9] and Stefania Sabella [1,*]

[1] Istituto Italiano Di Tecnologia, Nanoregulatory Platform, Drug Discovery and Development Department, Genova 16163, Italy; catherine.carnovale@gmail.com (C.C.); luisana.dicristo@iit.it (L.D.C.);
[2] Dipartimento di Chimica e Biologia "A. Zambelli", Università di Salerno, Via Giovanni Paolo II 132, Fisciano, 84084 Salerno, Italy; dguarnieri@unisa.it (D.G.);
[3] Research Centre for Biomaterials BIONAM, University of Salerno, Via Giovanni Paolo II 132, 84084 Fisciano, Italy
[4] Istituto Superiore di Sanità (ISS), Rome 00161, Italy; isabella.deangelis@iss.it (I.D.A.); flavia.barone@iss.it (F.B.)
[5] Laboratory of Chemistry and Biology of Metals (CBM), University Grenoble Alpes/CNRS/CEA, 38000 Grenoble, France; giulia.veronesi@cea.fr
[6] ESRF, the European Synchrotron, 71 Av. des Martyrs 38000 Grenoble, France
[7] Electron Microscopy Facility, Istituto Italiano di Tecnologia, Via Morego 30, 16163 Genova, Italy; alice.scarpellini@iit.it
[8] HiQ-Nano, s.r.l. Via Barsanti, Arnesano, 73010 Lecce, Italy; mariada.malvindi@hiqnano.com
[9] Nanobiointeractions & Nanodiagnostics, Istituto Italiano di Tecnologia (IIT), Via Morego 30, 16163 Genova, Italy; pierpaolo.pompa@iit.it
* Correspondence: stefania.sabella@iit.it
† These authors contributed equally to this work.

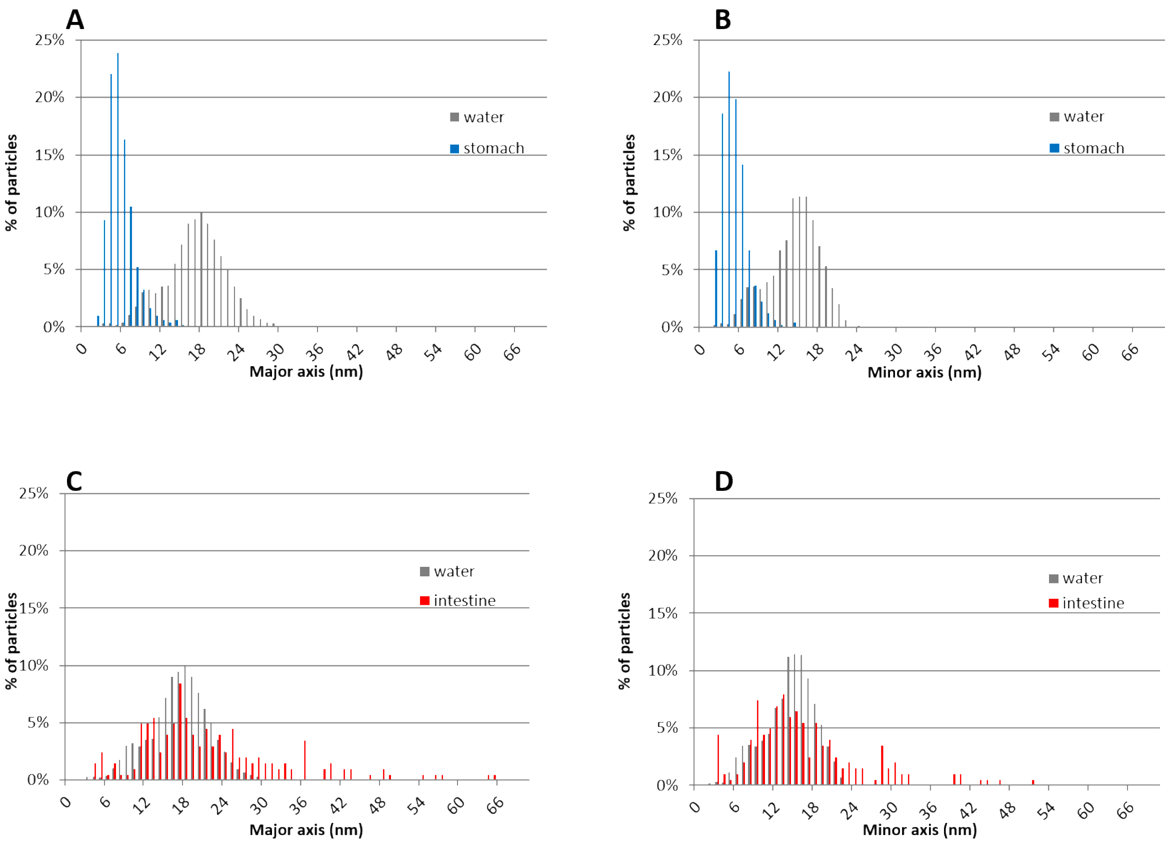

Figure S1. TEM Size distribution analysis of NM300K in water (grey), in the stomach compartment (dark blue) and the intestinal compartment (red); major axis (A, C) and minor axis (B,D). At least 500 particles for each TEM specimen were analysed by Image J (see method for details). Size: 18 nm ± 5 nm in the water; 5 nm ± 2 nm in the stomach and 17 nm ± 11 nm in the intestine compartments.

	CTRL charge (mV)	Saliva (mV)	Stomach (mV)	Intestine (mV)
**NM 300K**	−16.2	−35.0	−3.0	−32.0

Figure S2. Zeta potential of digested AgNPs measured after the application of the consecutive *in vitro* digestion assay.

Details of XRF analysis to reveal the presence of Ag close to the detection limit.

In order to understand whether Ag is present in cells outside the observed high-concentration regions (Ag rich spots) as Ag(I) ionic species, the average XRF signals of the cell area (excluding putative NPs) were extracted and observed in logarithmic scale (Main text Figure 3D,E, insets, red curves). No clear peak is observed in cells exposed to digested AgNPs (Figure 3D) around the energy of 2.984 eV (dashed vertical line), where the Ag $L_{\alpha 1}$ emission peak is expected. This means that diffused Ag signal within the cells, if present, is below the detection limit. In contrast, in cells exposed to undigested AgNPs, a clear peak centred at 2.984 eV is observed (Figure 3E, inset) in cells (red curve), but not in the resin background (green). The corresponding Ag concentration is estimated to 100 ppm by curve fitting, while Ag concentration in the putative NPs region of the same XRF map is estimated to 70,000 ppm (Figure 3E, black curve). This extremely wide range of detected Ag concentrations makes it impossible to visualize both putative AgNPs and diffused Ag in the same map, when Ag-dense regions are present.

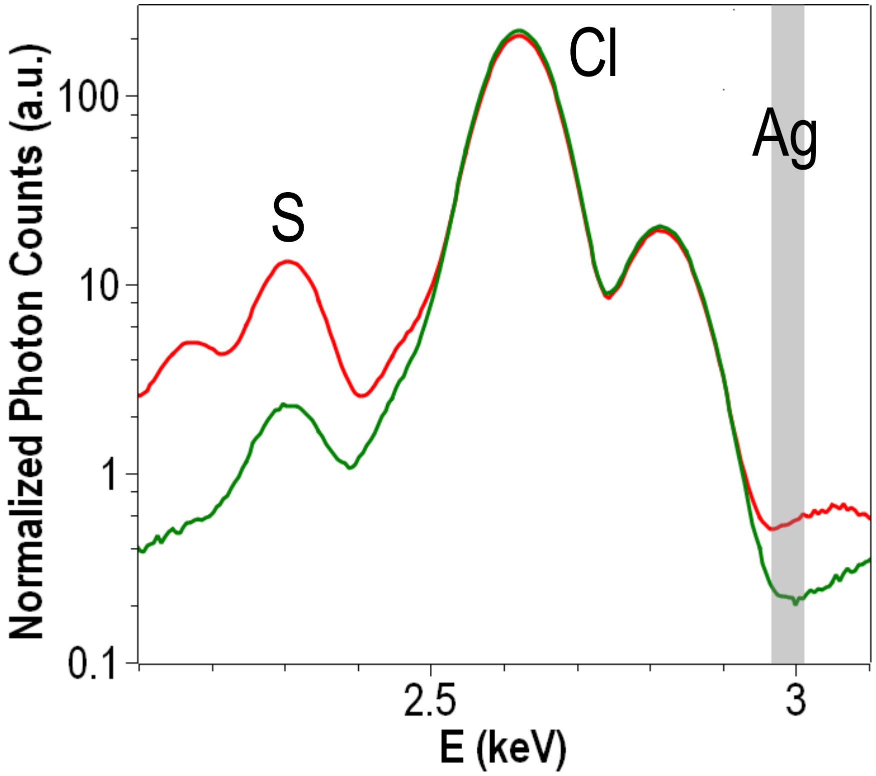

Figure S3. Sum XRF spectra of the cell region (red curve) and of cell-free resin (green curve) in epithelia exposed to dig Ag salt. The areas of interest were selected on the map reported in Figure 4C, and the sum spectra were normalized by the number of selected pixels. The energy region around 2.984 eV, where the Ag Lα1 emission peak would appear, is shadowed in grey.

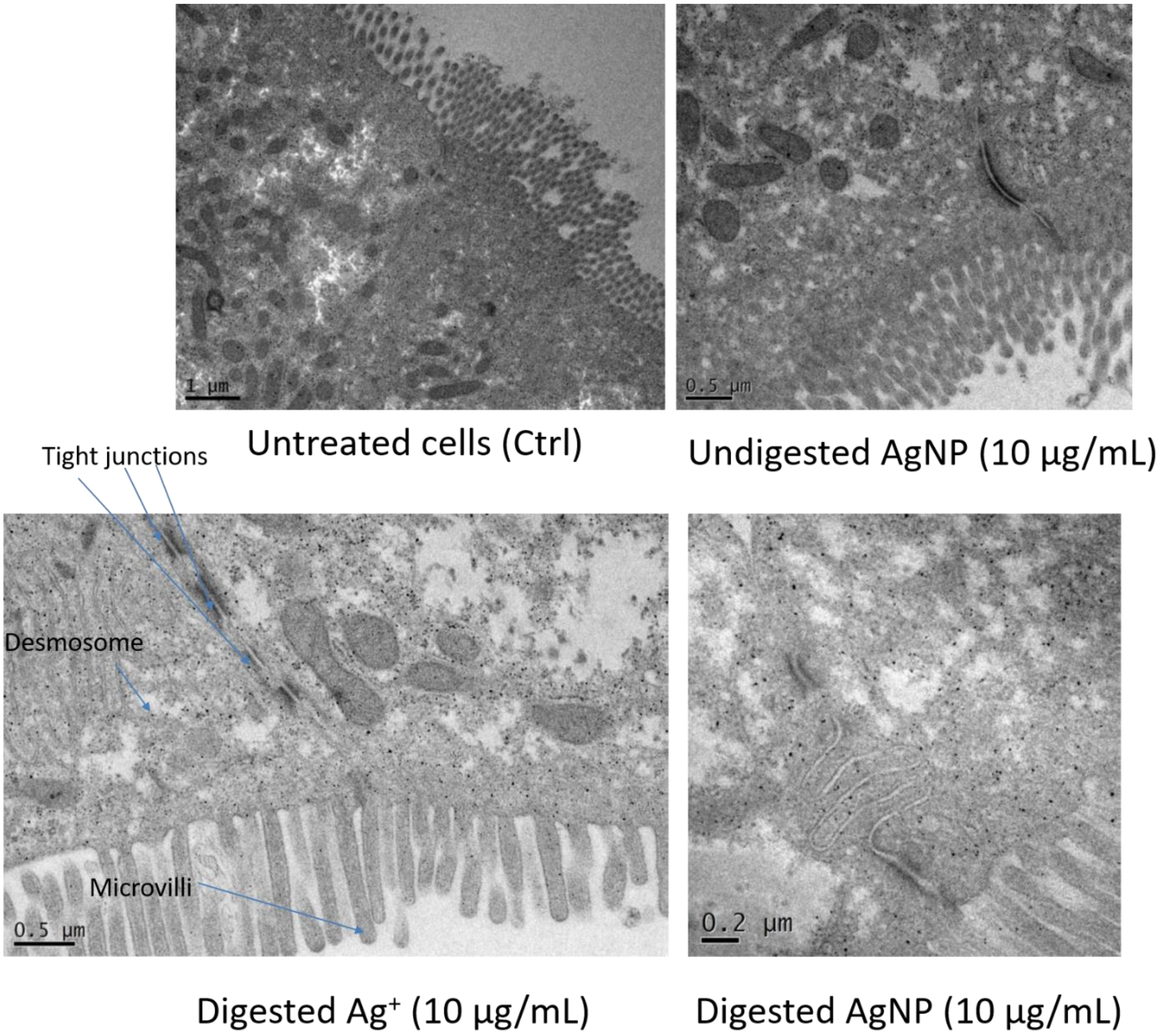

Figure S4. TEM image of Caco2 monolayer upon treatment with 10 μg/mL of undigAgNPs, digAgNPs, and dig Ag^+. Ctrl are untreated cells.

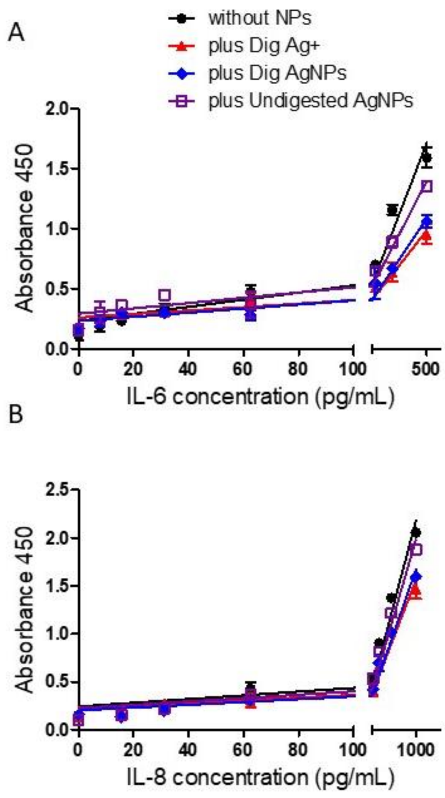

Figure S5. Representative calibration curve deriving from (A) IL-6 and (B) IL-8 standards dissolved in assay diluent with or without the addition of NPs implemented in the study.

Saliva	g/L
Inorganic	
KCl	0.90
KSCN	0.20
$NaH_2PO_4{\cdot}H_2O$	1.02
Na_2SO_4	0.57
NaCl	0.29
$NaHCO_3$	1.69
Organic/Protein	
urea	0.20
uric acid	0.015
mucin	0.025
Enzyme	
amylase	0.29

Table S6. Molecular composition of the saliva juice. The reported values are related to the final concentration used in the digestion assay.

Stomach	g/L
Inorganic	
NaCl	2.63
$NaH_2PO_4{\cdot}H_2O$	0.31
KCl	0.82
$CaCl_2$	0.31
NH_4Cl	0.31
Organic/Protein	
glucose	0.65
glucuronic acid	0.02
urea	0.085
glucosaminehydrochloride	0.33
BSA	1.00
mucin	3.00
Enzyme	
pepsin	2.50

Table S7. Molecular composition of the stomach juice. The reported values are related to the final concentration used in the digestion assay.

Duodenum	g/L
Inorganic	
NaCl	7.01
NaHCO3	3.39
KH2PO4	0.08
KCl	0.56
MgCl2.6H2O	0.05
CaCl2	0.15
Organic/Protein	
urea	0.10
BSA	1.00
Enzyme	
pancreatin	9.00
lipase	1.50

Table S8. Molecular composition of the duodenum juice. The reported values are related to the final concentration used in the digestion assay.

Bile	g/L
Inorganic	
NaCl	5.26
$NaHCO_3$	5.78
KCl	0.38
$CaCl_2$	0.17
Organic/Protein	
urea	0.25
BSA	1.80
Bile	30.00

Table S9. Molecular composition of the bile juice. The reported values are related to the final concentration used in the digestion assay.
